# Constitutive Oxidative Stress by SEPHS1 Deficiency Induces Endothelial Cell Dysfunction

**DOI:** 10.3390/ijms222111646

**Published:** 2021-10-28

**Authors:** Jisu Jung, Yoomin Kim, Jiwoon Na, Lu Qiao, Jeyoung Bang, Dongin Kwon, Tack-Jin Yoo, Donghyun Kang, Lark Kyun Kim, Bradley A. Carlson, Dolph L. Hatfield, Jin-Hong Kim, Byeong Jae Lee

**Affiliations:** 1 School of Biological Sciences, College of Natural Sciences, Seoul National University, Seoul 08826, Korea; tabris0520@snu.ac.kr (J.J.); foxmin12@snu.ac.kr (Y.K.); naji0708@snu.ac.kr (J.N.); qiaolu@snu.ac.kr (L.Q.); okkdi@snu.ac.kr (D.K.); yootackjin@snu.ac.kr (T.-J.Y.); kangd@snu.ac.kr (D.K.); 2 Interdisciplinary Program in Bioinformatics, College of Natural Sciences, Seoul National University, Seoul 08826, Korea; 880419@snu.ac.kr; 3 Severance Biomedical Science Institute, Graduate School of Medical Science, Brain Korea 21 Project, Gangnam Severance Hospital, Yonsei University College of Medicine, Seoul 06230, Korea; LKKIM@yuhs.ac; 4 Mouse Cancer Genetics Program, Center for Cancer Research, National Cancer Institute, National Institutes of Health, Bethesda, MD 20892, USA; carlsonb@dc37a.nci.nih.gov (B.A.C.); hatfielddolph@gmail.com (D.L.H.)

**Keywords:** selenium, selenoprotein, selenophosphate synthetase, endothelial cell, reactive oxygen species, cell growth, angiogenesis

## Abstract

The primary function of selenophosphate synthetase (SEPHS) is to catalyze the synthesis of selenophosphate that serves as a selenium donor during selenocysteine synthesis. In eukaryotes, there are two isoforms of SEPHS (SEPHS1 and SEPHS2). Between these two isoforms, only SEPHS2 is known to contain selenophosphate synthesis activity. To examine the function of SEPHS1 in endothelial cells, we introduced targeted null mutations to the gene for SEPHS1, *Sephs1,* in cultured mouse 2H11 endothelial cells. SEPHS1 deficiency in 2H11 cells resulted in the accumulation of superoxide and lipid peroxide, and reduction in nitric oxide. Superoxide accumulation in *Sephs1*-knockout 2H11 cells is due to the induction of xanthine oxidase and NADPH oxidase activity, and due to the decrease in superoxide dismutase 1 (SOD1) and 3 (SOD3). Superoxide accumulation in 2H11 cells also led to the inhibition of cell proliferation and angiogenic tube formation. *Sephs1*-knockout cells were arrested at G2/M phase and showed increased gamma H2AX foci. Angiogenic dysfunction in *Sephs1*-knockout cells is mediated by a reduction in nitric oxide and an increase in ROS. This study shows for the first time that superoxide was accumulated by SEPHS1 deficiency, leading to cell dysfunction through DNA damage and inhibition of cell proliferation.

## 1. Introduction

Selenium is an essential trace element that provides many health benefits. For example, selenium has been shown to prevent heart disease, have antiviral effects, and to boost the immune system, when it is consumed in adequate amounts, as discussed in [1] and references therein. This element also plays important roles in animal development and in the male reproductive system. Most of the beneficial effects of selenium are likely mediated by selenoproteins, which contain selenocysteine (Sec) at the active site [2]. Selenocysteine, the 21st amino acid in the genetic code, can be incorporated into a growing peptide in response to UGA codon translation [3,4]. Sec is produced by replacing the hydroxyl group of serine that is aminoacylated on tRNA^[Ser]Sec^ with inorganic selenium [2]. Selenophosphate serves as a selenium donor during Sec synthesis. Selenophosphate synthetase (SEPHS) catalyzes the reaction of selenophosphate synthesis from selenide at an ATP [5]. There are two isoforms of SEPHSs, SEPHS1 and 2, in eukaryotes, while only one form of SEPHS (SelD) exists in prokaryotes and Archaea. Mouse and human SEPHS1 are composed of 392 amino acids, and only two amino acid residues are different between human and mouse SEPHS1 at position 11 (serine in humans and threonine in mice) and 121 (methionine in humans and isoleucine in mice) according to the NCBI database (https://www.ncbi.nlm.nih.gov/protein/term=sephs1, accessed on 20 October 2021). These mammalian SEPHS1s have a high sequence homology with their SEPHS2s counterparts. SEPHS in *E. coli* (SelD) is composed of 347 amino acids (~37 kDa). The functions of prokaryotic SelD and eukaryotic SEPHS2 have been well established. SelD and SEPHS2 synthesize selenophosphate using inorganic selenium and ATP as substrates. The gamma phosphate of ATP is cleaved and attached to selenium to form selenophosphate. Interestingly, the beta phosphate on the remaining ADP is further cleaved, leaving free inorganic phosphate and AMP as final products [6]. Although SEPHS1s have high sequence homology with their SEPHS2 paralogues, they do not generate selenophosphate. It is of interest that SEPHS1 still retains the ability to cleave the gamma phosphate from ATP [7]. Furthermore, SEPHS1 plays an essential role in cell proliferation and survival. Knockout of the SEPHS1 gene, *Sephs1,* by P-element insertion in *Drosophila* resulted in embryonic lethality at the third instar larval/pupal stage [8]. When this gene was disrupted at the 5’-untranslated region, the imaginal disc was subject to aberrant formation. The cell number in mutant imaginal discs and in the brain was reduced, and apoptotic cells were observed in the abnormal disc. In another P-element mutant, the larval brain size was reduced, and DNA synthesis decreased significantly [9]. In *Drosophila* embryo-derived SL2 cells, the deficiency of SEPHS1 (SelD) led to a significant reduction in cell proliferation, and interestingly, also to the formation of megamitochondria [10]. These phenotypic changes occurred through the inhibition of pyridoxal phosphate synthesis. In mammals, SEPHS1 also plays key roles in cell proliferation and survival. In mice, systemic *Sephs1* knockout led to embryonic lethality. The knockout embryos were clearly underdeveloped by day E8.5 and virtually resorbed by day E14.5 [11]. Knockdown of *Sephs1* mRNA in both mouse and human cells also suppressed cell proliferation. Malignant properties, including cell invasion and foci formation, were inhibited by SEPHS1 deficiency in F9 cells [11], which are a mouse embryonic cancer cell line. Notably, accumulation of reactive oxygen species (ROS), especially hydrogen peroxide (H_2_O_2_), was observed in *Sephs1*-knockout F9 cells.

ROS include peroxides, superoxide, hydroxyl radicals, singlet oxygen, and alpha-oxygen species. Among these ROS, superoxide is frequently used as the precursor of most other ROS. Dismutation of superoxide produces H_2_O_2_. Partial reduction of H_2_O_2_ forms a hydroxide ion and a hydroxyl radical and full reduction of H_2_O_2_ produces water. Although ROS can be provided exogenously [12,13], it can also be produced endogenously. The cellular sources of ROS include electron transport chain complexes in mitochondria, as well as NADPH oxidase, the cytochrome P450 system and xanthine oxidoreductase (XOR) [14]. Among these, the electron chain complexes are the main source of ROS generation. Complex I and III produce superoxide, while complex IV produces H_2_O_2_. NADPH oxidases (NOXs) are a complex of enzymes that produce superoxide by oxidizing NADPH to NADP+. NOX was originally found in phagocytic cells and is highly expressed in immune cells. However, NOXs are also expressed in many other different tissues [15]. Four different NOX isoforms and two dual oxidases (DUOX) were discovered. These NOX family genes are expressed in a tissue-specific manner and localized in specific subcellular organelles. For example, endothelial cells express NOX1 and NOX4. NOX4 is localized in the mitochondria and produces H_2_O_2_ [16,17]. DUOXs are mainly expressed in thyroid cells, and also oxidize NADPH to produce NADP and protons, as well as producing H_2_O_2_. XOR has dual enzyme activity that produces xanthine from hypoxanthine and uric acid from xanthine. XOR can be reversibly converted into two different forms, xanthine dehydrogenase (XDH) and xanthine oxidase (XO) in mammals. Of these, XO uses oxygen to produce H_2_O_2_ and superoxide by transferring monovalent and divalent electrons to O_2_, respectively [18]. It is known that the ratio of XDH/XO is affected by cellular conditions. For example, in healthy tissues, the XDH form is most abundant, and this form uses NAD^+^ as a cofactor. However, in diseased cells, calcium-activated proteinases cleave XDH to XO, and XO uses oxygen as a cofactor [19,20]. It was also shown that the conversion of XDH to XO is accelerated under ischemic conditions [21]. 

The levels of ROS produced endogenously are tightly controlled by diverse ROS scavengers within the cell under normal conditions. Natural intracellular ROS scavengers include enzymes such as superoxide dismutases (SODs), catalases, glutathione peroxidases (GPXs), thioredoxin reductases (TXNRDs), and glutaredoxins (GLRXs). In normal cells, ROS can be used as a signaling molecule to activate cell proliferation and defense. However, various ROS species can yield cytotoxic effects, such as DNA damage and cell death, when the levels are high enough to cause oxidative stress [22]. 

Although SEPHS1 has been implicated in cell proliferation and oxidation/reduction homeostasis, the understanding of the detailed mechanism of how SEPHS1 functions is still limited. In our previous study, SEPHS1 deficiency was shown to inhibit cell growth and malignancy in F9 cancer cells [11]. It is well known that endothelial cells are involved in the formation of new blood vessels, i.e., angiogenesis. Therefore, we hypothesized that SEPHS1 deficiency in endothelial cells would reduce the ability of angiogenesis in tumors. A cultured endothelial cell line model is well suited to elucidate a more in depth understanding of the underlying mechanism(s) of SEPHS1 effects on angiogenesis. Since 2H11 is a commonly used cancer cell line derived from mouse endothelial cells, we chose 2H11 to investigate the function of SEPHS1 in endothelial cells. In this study, we examined the effect of SEPHS1 loss in endothelial cells by the targeted removal of *Sephs1,* and found that superoxide, not H_2_O_2_, was accumulated. We also observed that superoxide led to morphological changes of the cell through reducing focal adhesion, growth inhibition by oxidative-stress-mediated DNA damage, and loss of angiogenic ability by downregulating nitric oxide which was induced by oxidative stress.

## 2. Results

### 2.1. Sephs1 Knockout Leads to Morphological Changes

**Figure 1:**
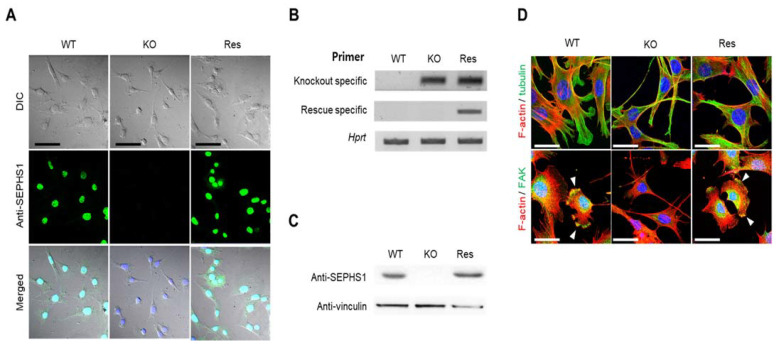
Construction of *Sephs1* knockout and rescue 2H11 endothelial cancer cells. Confirmation of *Sephs1* knockout by (**A**) immunocytochemistry (Scale bars represent 100 μm) and by (**B**) RT-PCR (the knockout-specific and rescue-specific primer sets are described in Materials and Methods) or (**C**) Western blot analysis. *Hprt* and anti-vinculin were used as internal controls. (**D**) Immunostaining of cytoskeletons and focal adhesion. Red and green color designate F-actin and α-tubulin, respectively (upper panel). In the lower panel, F-actin is in red and FAK in green. Arrowheads in the lower panel designate focal adhesion. Since most FAK is overlapped with F-actin, it appears as a yellow color. Scale bars represent 50 μm. DIC, digital image correlation; WT, wild type; Res, rescue; KO, knockout; FAK, focal adhesion kinase.

To study SEPHS1 function in endothelial cells, we constructed a *Sephs1*-knockout cell line targeting exon 8, using an endothelial cancer cell line 2H11, combined with CRISPR/Cas9 technology (see Figure S1 for the position of the target site). 2H11 is derived from an immortalized cell line established by transformation of mouse lymphoid endothelial cells with SV40 large T antigen. Among 14 independent clones that were puromycin-resistant, four possible mutant candidates were selected and subjected to sequencing to identify mutations. Interestingly, although positions and sequences of mutations were different among the clones, all knockout clones showed four different mutations each. For example, the knockout cell line 8-22 used in this study showed four different mutations, including a base insertion and deletions leading to a frameshift mutation in exon 8 (Figure S1). In addition to exon 8, we constructed knockout cell lines targeting exons 3 and 7. All the knockout clones also contained four different mutations in each single clone (data not shown). These results suggest that there are four copies of *Sephs1*. As shown in Figure 1, no SEPHS1 protein was detected by immunocytochemistry or Western blot analysis in the knockout cells (Figure 1A–C). To exclude off-target effects by the knockout, a rescue construct was produced by introducing silent mutations into the guide RNA target site. The levels of SEPHS1 expressed in this rescue cell line increased slightly compared with those of wild-type 2H11 cells, suggesting the rescue *Sephs1* construct recovered from the knockout effect. Unlike in other cell lines, such as the embryonic cancer F9 cell line in which SEPHS1 expression was ablated, knockout of *Sephs1* in 2H11 cells led to a morphological change from a fibroblast-like shape to a spindle shape with long, thin cytoplasm (Figure 1D). Additionally, the number of focal adhesions at lamellipodia was significantly reduced (lower panel of Figure 1D). For these reasons, the knockout cells appeared to have weak attachment abilities and exhibited a thinner and longer morphology.

### 2.2. Superoxide Is Accumulated by SEPHS1 Deficiency in Endothelial Cells

**Figure 2:**
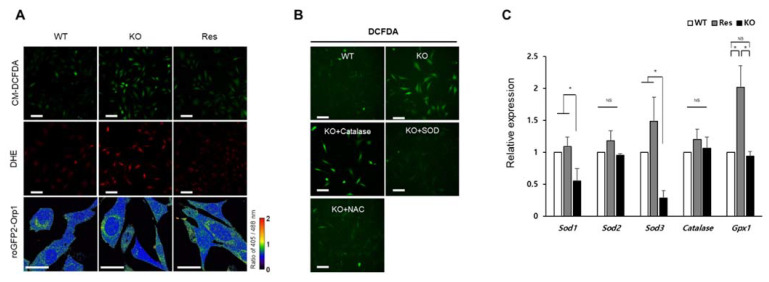
Identification of ROS type accumulated in *Sephs1*-knockout 2H11 cells. (**A**) Cells were stained with CM-DCFDA for general ROS and DHE for superoxide, and hydrogen peroxide levels were measured using roGFP2-Orp1 probe. The ratio between oxidized Orp (405 nm) and reduced Orp (488 nm) was calculated as described in Materials and Methods. Scale bars represent 100 μm for CM-DCFDA and DHE, and 50 μm for roGFP2-Orp1, respectively. (**B**) Confirmation of superoxide accumulation by treatment of scavengers. Scale bars represent 100 μm. (**C**) Measuring expression levels of ROS-scavenging enzymes by real-time PCR. The primers used in this experiment are shown in Table S1. NS, and * indicate not significant, and *p*-value < 0.05, respectively. WT, wild type; Res, rescue; KO, knockout.

In this study, the type of ROS accumulated in *Sephs1*-knockout cells was determined by staining with fluorescent dye or a GFP probe (Figure 2). Staining with CM-DCFDA showed that total ROS levels were increased by approximately 2.4-fold in *Sephs1*-knockout cells compared to wild-type controls (Figure 2A and Figure S2A). Furthermore, DHE staining demonstrated that superoxide levels were significantly increased (approximately 2.2-fold) in *Sephs1-knockout cells* (Figure 2A and Figure S2B). To detect the levels of cytosolic H_2_O_2_, cells were transfected with a roGFP-Orp1 probe [23,24], and both oxidized and reduced forms of Orp1 were measured. The ratio of the oxidized/reduced form was 0.96, 0.95, and 0.96 in wild-type control, knockout, and rescue cells, respectively (Figure S2C). This result clearly indicates that the levels of H_2_O_2_ were not changed by SEPHS1 deficiency in 2H11 cells. With these cytological data, we can conclude that superoxide, not H_2_O_2_, was accumulated by SEPHS1 deficiency in endothelial cells.

ROS can be accumulated in cells by overproduction of ROS and/or by reducing their scavengers. As shown in Figure 2B, supplementation of the cell culture medium with either SOD or N-acetyl cysteine (NAC) reduced intracellular ROS levels, while addition of catalase did not (Figure S2D). These data suggest that accumulation of superoxide in *Sephs1*-knockout cells was due to the lack of SOD and possibly other superoxide scavengers. This idea was confirmed by examining the RNA levels of ROS scavengers. As shown in Figure 2C, the RNA expressions of *Sod1* and *Sod3* decreased significantly in *Sephs1*-knockout cells. SOD1 destroys superoxides that are normally produced within the cells, while SOD3 catalyzes the dismutation of superoxide in the extracellular space secretion. However, the expression of enzymes such as catalase and glutathione peroxidase 1 (GPX1) that catalyze the reduction of H_2_O_2_*,* was not changed. These results indicate that superoxide accumulation in *Sephs1*-knockout endothelial cells is mediated by downregulating the expression of superoxide scavengers. Notably, *Sod2* levels were not decreased by *Sephs1* knockout. In addition, mitochondrial superoxide levels were not changed between wild-type and knockout cells when the cells were stained with MitoSOX^TM^ Red (data not shown). These data suggest that superoxides generated in mitochondria were converted to H_2_O_2_ immediately after being produced.

**Figure 3:**
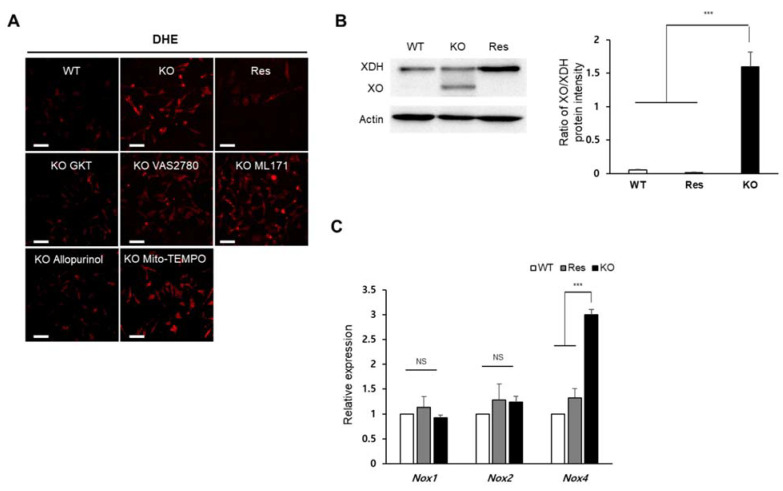
Identification of superoxide-generating sources in *Sephs1*-knockout 2H11 endothelial cells. (**A**) Cells were treated with selective inhibitors of superoxide production (allopurinol, GKT, VAS2780 and ML171) or superoxide scavenger (Mito-TEMPO). Scale bars represent 100 μm. (**B**) Western blot analysis using anti-XOR antibody. Actin was used as an internal control. XOR, xanthine oxidoreductase; XO, xanthine oxidase. (**C**) Measuring relative expression of NADPH oxidases by real-time PCR. Relative expression represents the ratio of ΔCt between wild type (WT) and knockout (KO) or rescue (Res) cells. NS and *** indicate not significant and *p*-value < 0.001, respectively.

To identify which synthesis pathway contributes to the accumulation of superoxide in the *Sephs1*-knockout 2H11 cells, cells were treated with chemicals that inhibit individual pathways, and superoxide levels were observed using DHE staining. As shown in Figure 3A, the addition of allopurinol (XO inhibitor) and GKT137831 (NOX1 and 4 inhibitor) decreased superoxide levels in *Sephs1*-knockout cells near to those levels observed in wild-type control cells (see also Figure S3). However, the treatment with VAS2780 (NOX2 inhibitor), ML171 (NOX1 inhibitor) or Mito-TEMPO (mitochondrial superoxide scavenger) did not reduce superoxide levels. These results suggest that XO and NOX4 are the major sources of superoxide production in *Sephs1*-knockout endothelial cells. The increase in superoxide levels caused by XO can be achieved by increasing the expression of XO. Generation of XO, which is produced by cleaving xanthine oxidoreductase (XOR), was dramatically induced by *Sephs1* knockout (Figure 3B). In *Sephs1*-knockout cells, the ratio between XOR and XO was approximately 1, while the XO form was not detectable in wild-type control and rescue cells. Although GKT137831 is an inhibitor of both NOX1 and NOX4, only *Nox4* mRNA levels were increased significantly by *Sephs1* knockout (Figure 3C). These results indicate that superoxide accumulation in *Sephs1*-knockout cells occurs both by a reduction in superoxide scavengers and by an increase in superoxide-producing enzymes such as XO and NOX4. 

### 2.3. SEPHS1 Regulates the Levels of Reactive Nitrogen Species and Lipid Peroxidation

**Figure 4:**
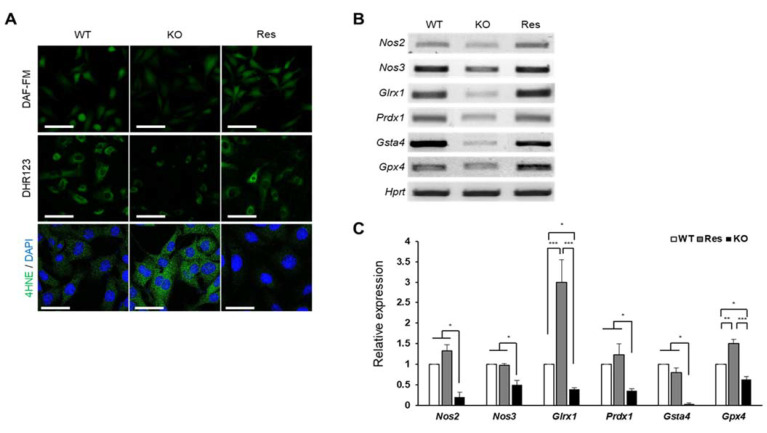
Effect of SEPHS1 deficiency on reactive nitrogen species generation and lipid peroxidation in 2H11 cells. (**A**) Staining of cells with fluorescent dyes to detect nitric oxide (DAF-FM), peroxynitrite (DHR123), and lipid peroxidation (4HNE). Scale bars in DAF-FM and DHR123 represent 100 μm, and 50 μm in in 4HNE. (**B**) Measuring expression levels of redox regulators participating in RNS generation and lipid oxidation by RT-PCR and (**C**) real-time PCR. *, ** and *** indicate *p*-value < 0.05, 0.01 and 0.001, respectively. The primers used in this experiment are shown in Table S1. WT, wild type; Res, rescue; KO, knockout.

In addition to the production of H_2_O_2_, superoxide can also be used as a substrate for the production of other free radicals and reactive species (FRRS) such as reactive nitrogen species (RNS) and peroxidated lipids. We examined how the accumulated superoxide affects the formation of these FRRS. As shown in Figure 4A, nitric oxide (NO) levels (DAF-FM) and peroxynitrite levels (DHR123) were significantly reduced in *Sephs1*-knockout cells (Figure S4A,B). In addition, the mRNA levels of nitric oxide synthase 2 and 3 (*Nos2* and *Nos3*) were also significantly reduced (Figure 4B,C). Since NO is a substrate of peroxynitrite production, the decrease in peroxynitrite levels in *Sephs1*-knockout cells is likely due to the shortage of a substrate. Conversely, the lipid peroxidation was dramatically increased (see 4HNE staining in Figure 4A and Figure S4C) suggesting that the lipids used as substrates for lipid peroxidation were sufficiently present to react with superoxide. In addition, we found that mRNA levels of scavengers of lipid peroxidation products such as glutaredoxin 1 (*Glrx1*), peroxiredoxin 1 (*Prdx1*), glutathione S-transferase a4 (*Gsta4*) and glutathione peroxidase 4 (*Gpx4*) were downregulated in the *Sephs1*-knockout cells (Figure 4B). Therefore, it seems that the lipid peroxidation in the *Sephs1*-knockout cells was induced by both sufficient substrate and a reduction in scavengers.

### 2.4. Superoxide Inhibits Cell Proliferation at G2/M Phase

**Figure 5:**
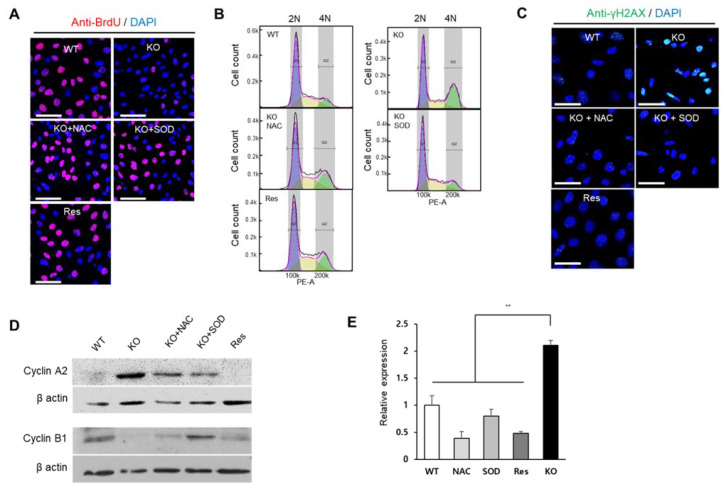
SEPHS1 deficiency affects cell proliferation, cell cycle and DNA damage in 2H11 cells. (**A**) Cell proliferation assay by BrdU incorporation. BrdU incorporation signal is in red, and the nucleus was counterstained with DAPI (blue). Scale bars represent 100 μm. (**B**) The effect of SEPHS1 deficiency on cell cycle progression. Cells were stained with PI and subjected to FACS analysis. DNA content of each cell was visualized as a histogram. 2N and 4N ploidy were grouped by interval gate. (**C**) Detection of DNA damage response by immunostaining with anti-gamma H2AX antibody. Nucleus was counterstained with DAPI. Scale bars represent 50 μm. (**D**) Expression pattern of G2/M arrest markers by Western blotting. (**E**) Measuring expression levels of GADD45β by real-time PCR. ** designates *p*-value < 0.01. WT, wild type; Res, rescue; KO, knockout.

In addition to ROS regulation, another common feature of SEPHS1 is that it is required for cell proliferation and viability [1]. BrdU incorporation assays to assess DNA synthesis revealed that cell proliferation was reduced by approximately 5-fold in *Sephs1*-knockout cells compared to that in wile-type control and rescue cells (Figure 5A and Figure S5A). BrdU incorporation was restored to normal levels by the addition of NAC or SOD, suggesting that accumulated superoxide was the main cause of the inhibition of cell proliferation. 

Flow cytometric analysis showed that the number of cells in the G2/M phase was significantly increased (approximately 2-fold) by SEPHS1 deficiency (Figure 5B and Figure S5B). However, the number of ROS scavenger-treated knockout cells arrested in G2/M was decreased similar to that of wile-type control and rescue cells (Figure 5B and Figure S5B). Generally, ROS affect cell cycle progression by causing DNA damage. For example, ROS induce the formation of gamma H2AX (a phosphorylated form of H2AX) foci that represent a double-strand DNA break marker, and subsequently leads to G2/M phase arrest [25,26]. As shown in Figure 5C, the number of gamma H2AX foci was increased approximately 3.5-fold in *Sephs1*-knockout cells compared to that of the wile-type control (Figure S5C). The phosphorylation levels of gamma H2AX were decreased in rescue cells and ROS scavenger-treated knockout cells. In addition, we further examined the levels of G2/M checkpoint markers such as cyclin A2 and B1, and growth-arrest and DNA-damage protein beta (GADD45β). As expected, the protein levels of cyclin A2 were increased, but cyclin B1 decreased in *Sephs1*-knockout cells and ROS scavenger-treated cells (Figure 5D), suggesting that *Sephs1*-knockout cells were arrested at the G2/M phase. The mRNA expression of GADD45β showed a similar pattern to cyclin A2, suggesting G2/M phase arrest occurred through DNA damage (Figure 5E). These results strongly suggest that the superoxide accumulated by *Sephs1* knockout resulted in gamma H2AX formation through a double-strand break, and that this DNA damage subsequently led to G2/M phase arrest. 

### 2.5. SEPHS1 Deficiency Inhibits Angiogenic Activity of Endothelial Cells

**Figure 6:**
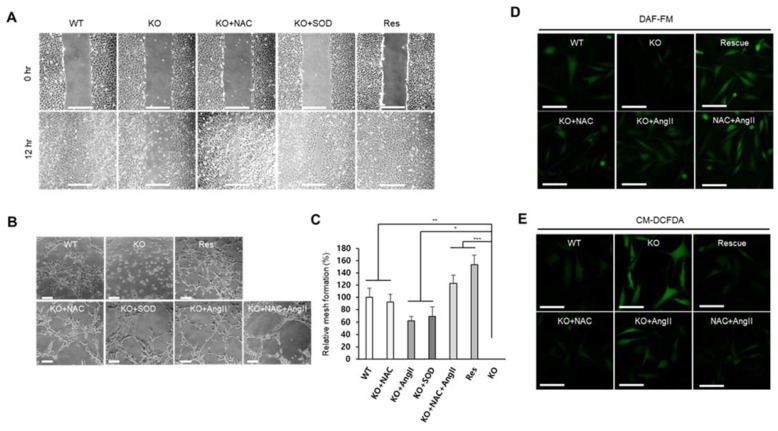
SEPHS1 deficiency impairs wound-healing ability and angiogenesis in 2H11 cells. (**A**) Wound-healing ability measured by scratch-wound assay. Scale bars represent 500 μm. (**B**) Tube formation assay to measure angiogenic ability. The images obtained by optical microscopy were analyzed using Angiogenesis Analyzer in ImageJ. Scale bars represent 100 μm. (**C**) Measurement of relative mesh formation. Mesh counts and relative mesh formation were obtained as described in Materials and Methods. *, ** and *** indicate *p*-value < 0.05, 0.01 and 0.001, respectively. (**D**) Detection of NO by staining with DAF-FM. Scale bars represent 100 μm. (**E**) ROS staining with CM-DCFDA. Scale bars represent 100 μm. WT, wild type; Res, rescue; KO, knockout.

Because endothelial cells are the main cell type of blood vessels, dysfunction of endothelial cells will cause the loss of angiogenic ability of an organism. Excessive ROS have been known to inactivate NO by oxidoreduction, and the reduction of NO causes endothelial dysfunction [27]. As described in the previous sections, the targeted removal of *Sephs1* in endothelial cells led to growth inhibition, reduction in focal adhesion, ROS accumulation and reduction in RNS levels. We, therefore, examined whether SEPHS1 deficiency affects the ability of endothelial cells to carry out angiogenesis. Cell migration is one of the important characteristics of angiogenesis. Wound healing is often used to measure migration ability of cells. As shown in Figure 6A, *Sephs1* knockout caused significant reduction in the endothelial cells wound-healing ability in scratch-wound assays. The wound-healing ability was reduced by approximately 2-fold in *Sephs1*-knockout cells compared to that in wild-type cells after 12 h incubation (Figure S6A). Both *Sephs1*-rescue- and ROS-scavenger (NAC and/or SOD)-treated *Sephs1*-knockout cells recovered wound-healing abilities with similar levels to those observed in wild-type 2H11 cells. This suggests that inhibition of wound-healing ability in *Sephs1*-knockout cells is mediated by superoxide accumulation. Future experiments to determine whether this inhibition of wound healing was due to the loss of migration ability or the inhibition of proliferation will require additional control experiments using a proliferation inhibitor such as aphidicolin. 

One of the commonly used methods for detecting angiogenic ability of endothelial cells is the tube formation assay [28]. As expected, targeted removal of *Sephs1* deprived the knockout cells of tube forming ability (Figure 6B). However, tube forming ability was recovered by the addition of NAC, SOD, or angiotensin II. Mesh formation is the last stage of tube formation. The number of meshes observed after NAC, SOD, angiotensin II, or NAC plus angiotensin II treatment of knockout cells was increased by 80%, 65%, 60% and 120%, respectively (Figure 6C). These data suggest that treatment of ROS scavengers or angiotensin II alone is not sufficient to recover angiogenic ability, but mixed treatment of a ROS scavenger and angiotensin II is enough for full recovery. Interestingly, tube formation in rescue cells was similarly increased compared with wile-type control cells (130%), suggesting that the ability of endothelial cells to carry out angiogenesis is correlated with the intracellular levels of SEPHS1 (Figure S6A–C). Since angiotensin II is an inducer of NO synthesis, NO levels were examined after treatment of angiotensin II in the knockout cells. As shown in Figure 6D, angiotensin II treatment increased NO levels in *Sephs1*-knockout cells similarly to those of wild type cells. Unexpectedly, the levels were also increased in the cells by NAC treatment. Treatment of NAC combined with angiotensin II increased NO level more than wild-type, and at similar levels to that of rescue cells. ROS levels were also examined after treatment of NAC and/or angiotensin II. The levels of ROS were negatively correlated to NO levels, suggesting ROS affects NO synthesis (Figure 6E). Notably, angiotensin II treatment reduced the ROS levels in the *Sephs1*-knockout cells, although not as much as with NAC treatment. Since regulation of intracellular NO and ROS levels determines the angiogenic ability in endothelial cells, these results suggest that SEPHS1 plays an essential role in angiogenesis by regulating the NO and ROS levels in endothelial cells.

## 3. Discussion

Although previous studies showed that the deficiency of SEPHS1 led to the accumulation of ROS, the types of ROS were not determined [10]. Recently it was reported that H_2_O_2_ was accumulated in *Sephs1*-knockout, F9, embryonic carcinoma cells [11]. In the *Sephs1*-knockout F9 cells, the expression of redox-homeostasis-related genes encoding such proteins as GLRX1 and various GSTs was dysregulated [11]. However, the levels of SODs and catalases were not changed by SEPHS1 deficiency in F9 cells, suggesting that H_2_O_2_ was accumulated mainly by dysregulation of genes involved in redox homeostasis. In this study, we found that superoxide, rather than H_2_O_2_, was accumulated in *Sephs1*-knockout 2H11 endothelial cancer cells, indicating a cell-type specificity for ROS types controlled by SEPHS1. As shown in the NIH database (https://www.ncbi.nlm.nih.gov/gene/22929, accessed on 20 October 2021), there is low cell-type specificity in *Sephs1* expression. The accumulation of different kinds of ROS in different cell types is, therefore, not likely dependent on the expression levels of *Sephs1*, but is likely dependent on the interaction of SEPHS1 with a different set of cellular components. It appears that SEPHS1 interacts with different ROS scavengers and/or producers depending on the cell type, since the expression levels of genes participating in oxidation/reduction homeostasis appear to differ. The mechanism of how SEPHS1 interacts with and regulates those proteins needs to be defined. It is interesting that among six pathways that produce superoxide, only XO and NOXs are the main sources of superoxide production in *Sephs1*-knockout endothelial cells. In the case of XO, deficiency of SEPHS1 induced the processing of XOR to produce XO, and enzymatic activity of XO was increased accordingly. Another important feature of *Sephs1*-knockout endothelial cells is the downregulation of *Sod1* and *Sod3,* which are localized in the cytoplasm. SOD2 expression was not decreased by SEPHS1 deficiency, suggesting that the mitochondrial electron transfer chain did not release superoxide into the cytoplasm. In conclusion, superoxide accumulation in *Sephs1*-knockout endothelial cells occurs both by increasing the superoxide-producing system and by reducing SOD expression.

**Figure 7:**
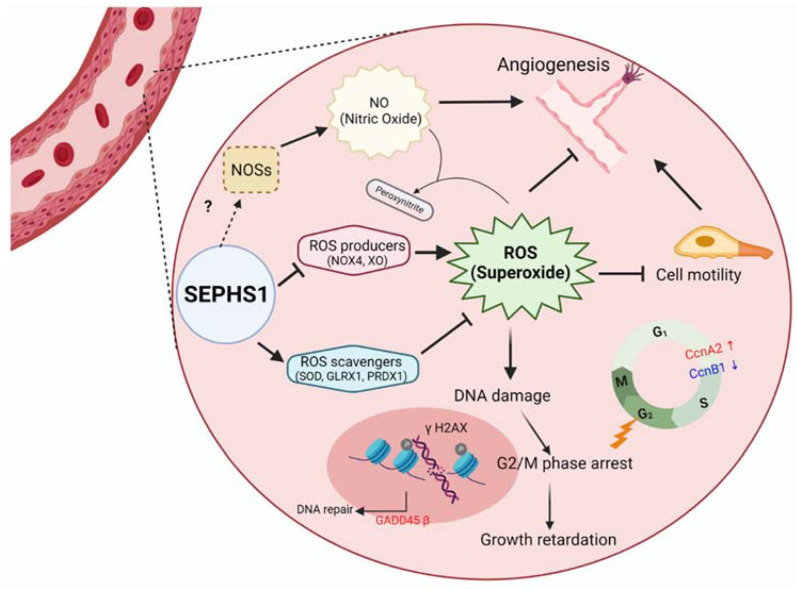
A schematic model for SEPHS1 function in mammalian endothelial cells. The cell represents an endothelial cell in the blood vessel. In normal conditions, SEPHS1 expresses ROS scavengers at appropriate levels, but inhibits superoxide generators such as NOX4 and XO. Superoxide is accumulated when SEPHS1 is deficient, and the superoxide causes endothelial cell dysfunctions such as growth retardation by G2/M phase arrest and loss of angiogenic ability by a decrease in NO levels. NO levels are decreased by downregulation of NOSs. Oxidative stress by superoxide accumulation leads to DNA damage and then G2/M phase arrest. The mechanism of how SEPHS1 inhibits the expression of NOSs is unclear. Arrows (→) designate activation and barred-lines (⊥) designate inhibition.

Although the accumulated ROS types are different in *Sephs1*-knockout cells depending on cell type, cell proliferation is commonly inhibited. *Sephs1*-knockout endothelial cells were arrested at the G2/M phase, and phosphorylation of H2AX was increased by ROS accumulation. This modification of H2AX is a known marker of double-strand DNA breaks [29]. Therefore, it is highly likely that the superoxide accumulated in the cell induces DNA damage and arrests cells at the G2/M checkpoint (Figure 7). 

Superoxide can induce the production of reactive nitrogen species such as peroxynitrite and lipid peroxy radicals. In *Sephs1*-knockout endothelial cells, only lipid peroxidation was induced and, unexpectedly, peroxynitrite production was inhibited through the inhibition of NO production. These reduced NO levels were due to the downregulation of NOSs. Superoxide accumulation in endothelial cells seems to lead to the inhibition of NO levels, because NO levels could be increased by ROS scavenger treatment [29]. The mechanism of how SEPHS1 deficiency inhibits the expression of NOSs is unclear.

Endothelial cells form the lining of the interior surface of blood and lymphatic vessels. Therefore, endothelial cells play key barrier roles between vessels and neighboring tissues and in controlling the flow of blood and lymph. In this study, we revealed that SEPHS1 plays an important role in maintaining NO levels in endothelial cells, possibly by regulating ROS homeostasis. Furthermore, we showed that NO is not the only factor for angiogenic functions of endothelial cells, but that other factor(s) is (are) required for SEPHS1-mediated angiogenesis. Since angiogenesis is the most prominent feature of tumor growth, and since we have previously shown that SEPHS1 deficiency inhibits tumor-cell malignancy [11], targeted removal of SEPHS1 in endothelial cells may provide a potential new measure for antitumor therapy.

## 4. Materials and Methods

### 4.1. Materials

Dulbecco’s modified Eagle’s medium (DMEM) was purchased from Hyclone (Logan, UT, USA). Fetal bovine serum (FBS) was purchased from Serana (Bunbury, Australia). Antibiotic–antimycotic, Dulbecco’s phosphate-buffered saline (PBS), trypan blue solution, rhodamine phalloidin, Lipofectamine^®^ Reagent, blasticidin S HCl and puromycin were purchased from Life Technologies (Waltham, MA, USA). Neomycin was purchased from AG Scientific (San Diego, CA, USA). LentiCRISPR v2 and psPAX2 were purchased from Addgene (Watertown, MA, USA). Superoxide dismutase, catalase, N-acetyl cysteine (NAC), apocynin, allopurinol, Mito-TEMPO, angiotensin II, dihydroethidium (DHE), dihydrorhodamine 123 (DHR123), propidium iodide (PI), PMSF cocktail (protease inhibitor) and DAPI were purchased from Sigma (St. Louis, MI, USA). GKT137831 was purchased from Cayman (Ann Arbor, MI, USA). 5-(and-6)-chloromethyl-2′,7′-dichlorodihydrofluorescein diacetate, acetyl ester (CM-DCFDA) and diaminofluorescein-FM diacetate (DAF-FM) were purchased from Molecular Probes (Eugene, OR, USA). 5-Bromo-2′-deoxy-uridine (BrdU) Labeling and Detection Kit was purchased from Roche (Basel, Switzerland). ECL reagent was purchased from Amersham (Buckinghamshire, UK). Antibodies against SEPHS1(sc-365945), BrdU(sc-32323), gamma H2AX(sc-517348), Cyclin B1(sc-245), Xanthine oxidase(sc-398548), CFL488 conjugated mouse IgG(sc-533653) and CFL488 conjugated rabbit IgG(sc-516248) were purchased from Santa Cruz Biotech (Dallas, TX, USA). Anti-alpha tubulin(ab15246), anti-4HNE(ab46545), anti-cyclin A2(ab137769), anti-beta actin (ab8227), antimouse and rabbit IgG cy3(ab97035 and ab97075) antibodies were purchased from Abcam (Cambridge, UK). HRP conjugated anti-rabbit IgG and HRP conjugated anti-mouse IgG(GTX213111-01) were purchased from Genetex (Irvine, CA, USA). Matrigel used for tube formation assay was purchased from Corning (Corning, NY, USA). MG^TM^ Tissue SV kit was purchased from MG Med, (Seoul, Korea). PVDF membranes was purchased from GE Healthcare (Chicago, IL, USA) FlowJo™ Software Version 10.8.1 from BD (Ashland, OR, USA).

### 4.2. Cell Culture

The 2H11, HEK293T and GP2-293 cells were cultured as described previously with minor modifications [11]. Cells were incubated in DMEM with 10% FBS and 1% antibiotic–antimycotic in a humidified atmosphere containing 5% CO_2_ at 37 °C.

### 4.3. CRISPR-Based Knockout Cell Line Construction 

sgRNA (single guide RNA sequences) targeting a region in exon 8 of *Mus musculus Sephs1* was designed using the CRISPR online design tool (http://crispr.mit.edu, accessed on 26 September 2021). The sequences of sgRNA_E8 were: 5′-CACCGTAGGCCGAACATGTTTCCGC-3′/5′-AAACGCGGAAACATGTTCGGCCTAC-3′. These complementary oligonucleotides of sgRNA were annealed and cloned into LentiCRISPR v2 vector as described previously [30]. 

For lentivirus production, HEK293T cells were transfected with both the constructed sgRNA-containing LentiCRISPR v2 vector and virus packaging plasmid psPAX2 and pMD2.G. After a 48hr incubation, lentiviruses were harvested by filtration through 0.45 µm filter. 2H11 cells were infected with the harvested lentiviruses by incubating for 48hr, and then the infected cells were selected with 2 mg/mL of puromycin for a further 6 days. Single clones were obtained using a 96-well plate. From each clone, genomic DNA was extracted and subjected to PCR amplification using primers (forward: 5′-ACAAAGT GGGTGTTGGGTGT-3′; reverse: 5′-AGCCTTGTAACCATCCTGCC-3′). The amplified DNA fragments were cloned into TA-cloning vector, and then each clone was sequenced. The knockout cell line was further confirmed by PCR using the primer set (forward: 5′-AAGCATGTGGCAATATGTTTGGAT-3′, reverse: 5′-GTGGCACCAGGTGTGGG-3′).

### 4.4. CRISPR-Based Rescue Cell Line Construction

To exclude any off-target effect of the *Sephs1* gene knockout, a rescue cell line was constructed as follows. First, silent mutations in sgRNA regions (see Figure S1) were introduced to wild-type *Sephs1* which were resistant to Cas9 cleavage and expressed wild-type SEPHS1 proteins. Site-directed mutagenesis was carried out by two-step PCR methods with primer sets (primer set 1 forward: 5’-TACCGAGCTCGGATCCGAAC-3’, reverse: 5’-CAATCCAAACATATTGCCACATGCTTTGCTCACAGCGGCCAT-3’; primer set 2 forward: 5’-CATGTGGCAATATGTTTGGATTGATGCATGGGACCTGCCAGA-3’, reverse: 5’-GGTTTAAACGGGCCCTCTAG-3’. The PCR products were cloned into a retroviral vector at the BamHI/EcoRI site (pRv.neo; [31]), and the plasmid was delivered to retroviral packaging cells (GP2-293). After incubating for 48 h, viral particles were harvested and used to infect 2H11 cells where *Sephs1* was knocked out. After infection, a rescue cell line was selected with G418 (400 µg/mL) and confirmed by PCR with primer set (forward: 5′-GCATTCCCCACAAAGGCAA-3′, reverse: 5′-AGCAAAGCCTGACACCCA T-3′), Western blot analysis and immunocytochemistry.

### 4.5. Real-Time PCR

Real-time PCR was performed as described previously [11] with minor modifications. Total RNA was isolated using TRIZOL reagent. Total RNA (2000 ng) was reverse-transcribed by Mo-MuLV reverse transcriptase, and real-time PCR was performed in triplicate using PowerUp^TM^ SYBR^TM^ Green Master Mix and Prism7300 (Applied Biosystems) according to the manufacturer’s instructions. Sequences of primers used in this study are provided in Table S1. The annealing temperature was set as 3 °C below the T_m_ of the primer set. The *Hprt* gene was used as an internal control.

### 4.6. Western Blot Analysis

Western blot analysis was carried out as described previously [11,32] with slight modifications. Briefly, cells were washed twice with PBS and harvested in ice-cold lysis buffer (PBS with 0.5% Triton X-100 and 0.1% PMSF cocktail). The protein concentrations of the resulting cell extracts were measured by Bradford dye-binding method and 20 μg of total protein from each sample were subjected to 10% SDS-polyacrylamide gel electrophoresis, then transferred to PVDF membranes. The membranes were incubated overnight at 4°C with primary antibodies against SEPHS1 (1:1000), vinculin, xanthine oxidase (1:1000 each), actin (1:5000), cyclin A2 (1:1000) or cyclin B1 (1:2000). Membranes were washed with Tris-buffered saline (TBS) containing 0.1% Tween 20 and incubated with secondary antibodies for 30 min at room temperature. Immunolabeling was detected using ECL reagent, and luminescence signal was detected using Chemi-Doc (Luminograph II, ATTO). The band intensities on each blot were quantified using ImageJ software (NIH).

### 4.7. Immunocytochemistry

Immunocytochemistry was carried out as described previously [10], with modifications. Briefly, 1.5 × 10^4^ cells were seeded on a 9 mm coverslip and fixed with 4% paraformaldehyde in PBS, washed with PBS twice, and then permeabilized with 0.1% Triton X-100 in PBS for 10 min. The permeabilized cells were blocked with 5% FBS in PBS for 1 h at room temperature and then incubated with primary antibodies with appropriate dilution folds; anti-SEPHS1 (1:100), anti-alpha-tubulin (1:200), anti-4-hydroxynonenal (4-HNE) (1:50), anti-BrdU (1:100), anti-gamma H2AX (1:100), and anti-CD31(1:50), respectively. Primary antibody binding was performed at 4 °C overnight, and then secondary antibody conjugated with fluorescent dye; anti-rabbit IgG conjugated with Cy3 and anti-mouse IgG conjugated with CFL488 was incubated (1:100) for 30 min at room temperature. Cells were observed by Diaphot 300 fluorescence microscope (Nikon FL, Tokyo, Japan) or LSM700 confocal microscope (Carl Zeiss, Jena, Germany). 

### 4.8. Determining ROS Types 

The detection of intracellular ROS was carried out with CM-DCFDA as described previously [10] with minor modifications. 2H11 cells were seeded at a density of 5 × 10^4^ cells/well in a 12-well plate 1 day before staining. The cells were incubated with 5 μM CM-DCFDA in DMEM containing 1% antibiotic–antimycotic without FBS for 30 min at 37 °C in 5% CO_2_, washed twice with PBS, and then observed under fluorescence microscope (Nikon FL) at an excitation wavelength of 470 nm.

Superoxide was stained with DHE as described previously [11], with slight modifications. Then, 5 × 10^4^ cells were prepared as above and stained by incubating cells in 10 μM DHE in DMEM containing 1% antibiotic–antimycotic and 10% FBS for 15 min at 37 °C. After washing with PBS, the fluorescence signals were observed under fluorescence microscope (Nikon FL) at an excitation wavelength of 531 nm.

Detection of hydrogen peroxide was carried out as described previously [11], with minor modifications. The cytosolic roGFP2-Orp1 vector [23,24] was transfected into each cell. After incubation for 24 h at 37 °C in 5% CO_2_, 1.5 × 10^4^ cells were seeded on a 9 mm coverslip and incubated for 12 h, washed with PBS, fixed with 4% paraformaldehyde in PBS and then observed under an LSM 700 confocal microscope (Carl Zeiss). The ratio between the oxidized (405 nm) and reduced (488 nm) forms of the probes was calculated for each cell according to Morgan et al. [23]. The intensities of the 405 nm and 488 nm image from the same original field (100× magnification) were obtained separately as described [11]. The intensity of the 405 nm images was divided by the intensity of the 488 nm images to calculate the ratio. This procedure was repeated in six different fields for each cell line, and the ratio images were created by dividing the 405 nm image by the 488 nm image pixel by pixel. The ImageJ ‘Blue Green Red’ Look Up Table (LUT) was used for creating false-color ratio pictures. 

### 4.9. Measurement of ROS Levels with Fluorescence Activated Cell Sorting

Cells were seeded at 2 × 10^5^ cells per well in a 6-well plate. On the following day, cells were stained with a ROS probe. After staining, cells were harvested and intracellular fluorescence intensity of the probe was quantified using a fluorescence-activated cell sorter (FACS, Canto II, BD Biosciences, Franklin Lakes, NJ, USA). A minimum of 2 × 10^4^ cells were counted from each sample and fluorescence distribution of cells was analyzed and displayed as a histogram.

### 4.10. Scratch-Wound Assay

Scratch-wound assay was carried out as described previously [31], with minor modifications. Cells were seeded at a density of 3 × 10^5^ cells per well in a 6-well plate. On the following day, cells were scratched with a yellow pipette tip, washed with PBS twice, and then further incubated in DMEM with 10% FBS at 37 °C for 12 h. The movement of cells into the wound area was measured after photographing the cells. The covered area was calculated by subtracting initial wound area from the remaining wound area at the end of the assay. To avoid scratch-width variation, the relative covered area (RCA) was calculated by RCA[%] = covered area × 100 [%]/initial area. Experiments were performed in triplicate. 

### 4.11. Tube Formation Assay

Tube formation assay was carried out as described in Cao et al. [33] with modifications. Cells were seeded at a density of 1.5 × 10^5^ cells per well in a 6-well plate. The cells were incubated with trypsin EDTA, neutralized with DMEM with 10% FBS and 1% antibiotic–antimycotic, and harvested by centrifugation. Cells were washed and resuspended with serum-free DMEM. Cells were then seeded at a density of 3 × 10^4^ cells per well in a Matrigel-precoated 96-well plate. A Matrigel-precoated 96-well plate was prepared a day before use by incubating the plate with 100 μL of Matrigel per well for 1 h at 37 °C. After incubating cells for 6 h at 37 °C, tube formations were observed with an optical microscope. The extent of tube formation was analyzed using “Angiogenesis Analyzer” software plugin for ImageJ as described previously [28].

### 4.12. Administration of ROS Scavengers and Inhibitor Treatments

ROS scavengers were treated as described by Kate et al. [34] with minor modifications. In this study, NAC, catalase, SOD and Mito-TEMPO were used in a concentration of 1 mM, 300 units/mL, 300 units/mL, and 50 μM, respectively. Selective inhibitors for xanthine oxidase and NADPH oxidases were administrated according to Augsburger et al. [35]. In this study, allopurinol, GKT136901, VAS2780, and ML171 were used at a concentration of 50 µM, 50 µM, 5 µM, and 5 µM, respectively. ROS scavengers and selective inhibitors were administrated overnight, and on the next day, cells were stained with an appropriate ROS probe observed with fluorescent microscope or confocal microscope.

### 4.13. Detection of Reactive Nitrogen Species

Reactive nitrogen species (RNS) analysis was carried out as described by Handa et al. [36] with modifications. For the detection of nitric oxide, cells were stained by incubating with 5 µM at 37 °C for 30 min. Peroxynitrite was detected by staining cells with DHR123, 10 µM at 37 °C for 30 min. Stained cells were observed with a fluorescence microscope (Nikon FL). The intensity of the RNS signal was quantified using FACS (Canto II, BD Biosciences) and FlowJo software.

### 4.14. Cell Cycle Analysis

Cell cycle analysis was performed by measuring DNA content after staining cells with propionic iodide (PI) [37]. Cells were seeded onto a 12-well plate at a density of 3 × 10^4^ cells/well. After a 12 h incubation, ROS scavengers such as SOD or NAC were administered into *Sephs1*-knockout cell overnight. On the following day, cells were harvested, washed once with PBS, and fixed in 70% ethanol at 4 °C overnight. The fixed cells were washed with PBS twice, resuspended with PBS containing RNase A (200 µg/mL) and incubated for 10 min at room temperature. Cells were then further incubated with 100 µg/mL of PI in PBS for 30 min at room temperature. Stained cells were analyzed using FACS (Canto II, BD Biosciences) and FlowJo software.

### 4.15. BrdU Incorporation Assay

BrdU incorporation assay was carried out using BrdU Labeling and Detection Kit (Roche) according to the manufacturer’s instructions. Briefly, cells were seeded onto a 24-well plate at a density of 1.5 × 10^4^ cells per well overnight. On the following day, BrdU was added to the growth media at the concentration of 10 µg/mL for 2 h. Incorporated BrdU was detected by immunocytochemistry using anti-BrdU antibody, and the fluorescent signals conjugated to the secondary antibody were observed using a confocal microscope (Carl Zeiss).

### 4.16. Statistics

Each experiment was performed in biological triplicate for statistical analysis. Statistical significance was tested by one-way ANOVA followed by Tukey’s multiple comparison test.

## Data Availability

All data are contained within the article or Supplementary Material are available for anyone to utilize without violating participant confidentiality. Additional information can be requested from authors upon reasonable request.

## Supplementary Materials

The following are available online at https://www.mdpi.com/article/10.3390/ijms222111646/s1, Figure S1: Sequences of sgRNA, mutations in a knockout cell line (8–22) and rescue construct, Figure S2: Quantification of intensity of ROS probes. Figure S3: Quantification of superoxide accumulation in scavenger and selective inhibitor treated cells. Figure S4: Measurement of signal intensities of RNS probe and 4-HNE. Figure S5. Analysis of cell proliferation. Figure S6: Measurement of cell motility and angiogenesis. Table S1: Primer sequences used in this study.
