# Supplementary material for: Constitutive Oxidative Stress by SEPHS1 Deficiency Induces Endothelial Cell Dysfunction
Source: Int J Mol Sci. 2021 Oct 28;22(21):11646. doi: 10.3390/ijms222111646 (PMC8584027; doi:10.3390/ijms222111646)
Supplement: Supplementary file 1 [file ijms-22-11646-s001.zip › ijms-1417096-supplementary.pdf]

```
        1317
WT      TGGCGAAGATGGCCGCTGTGAGCAAAGCCTGC-GGAAACATGTTCGGCCTAATGCATGGGACCTGCCCAGAGACGTCAGGTA
KO #1   TGGCGAAGATGGCCGCTGTGAGCAAAGCCTGC----AACATGTTCGGCCTAATGCATGGGACCTGCCCAGAGACGTCAGGTA
KO #2   TGGCGAAGATGGCCGCTGTGAGCAAAGCCTGCTCTTTACATGTTCGGCCTAATGCATGGGACCTGCCCAGAGACGTCAGGTA
KO #3   TGGCGAAGATGGCCGCTGTGAGCAAAGCCTGC--GAAACATGTTCGGCCTAATGCATGGGACCTGCCCAGAGACGTCAGGTA
KO #4   TGGCGAAGATGGCCGCTGTGAGCAAAGCCTG---------------------------------------------------
Res     TGGCGAAGATGGCCGCTGTGAGCAAAGCATGT-GGCAATATGTTTGGATTGATGCATGGGACCTGCCCAGAGACGTCAGGTA

WT      AAAAGGGCGGGGTGGGTGCTGTTCCCTGGCCTGAGGGTTCAAGTCTGAGCCTGACACCCATGCCTTCCCCCTGCTGGGAGTT
KO #1   AAAAGGGCGGGGTGGGTGCTGTTCCCTGGCCTGAGGGTTCAAGTCTGAGCCTGACACCCATGCCTTCCCCCTGCTGGGAGTT
KO #2   AAAAGGGCGGGGTGGGTGCTGTTCCCTGGCCTGAGGGTTCAAGTCTGAGCCTGACACCCATGCCTTCCCCCTGCTGGGAGTT
KO #3   AAAAGGGCGGGGTGGGTGCTGTTCCCTGGCCTGAGGGTTCAAGTCTGAGCCTGACACCCATGCCTTCCCCCTGCTGGGAGTT
KO #4   -----------------------------------------------------ACACCCATGCCTTCCCC-TGCTGGGAGTT
Res     AAAAGGGCGGGGTGGGTGCTGTTCCCTGGCCTGAGGGTTCAAGTCTGAGCCTGACACCCATGCCTTCCCCCTGCTGGGAGTT
```

Figure S1. Sequences of sgRNA, mutations in the knockout cell line (8-22), and rescue constructs. Genetic information of the cell line used in this study was visualized in an align format of the *Sephs1* coding sequence (from 1317). The PAM sequence is underlined. sgRNA is marked with a box. Deletion mutations in the KO cell line are marked as a hyphen, insertions are shown as bold-faced letters, and substitution mutations in rescue are shown in bold-faced red letters.

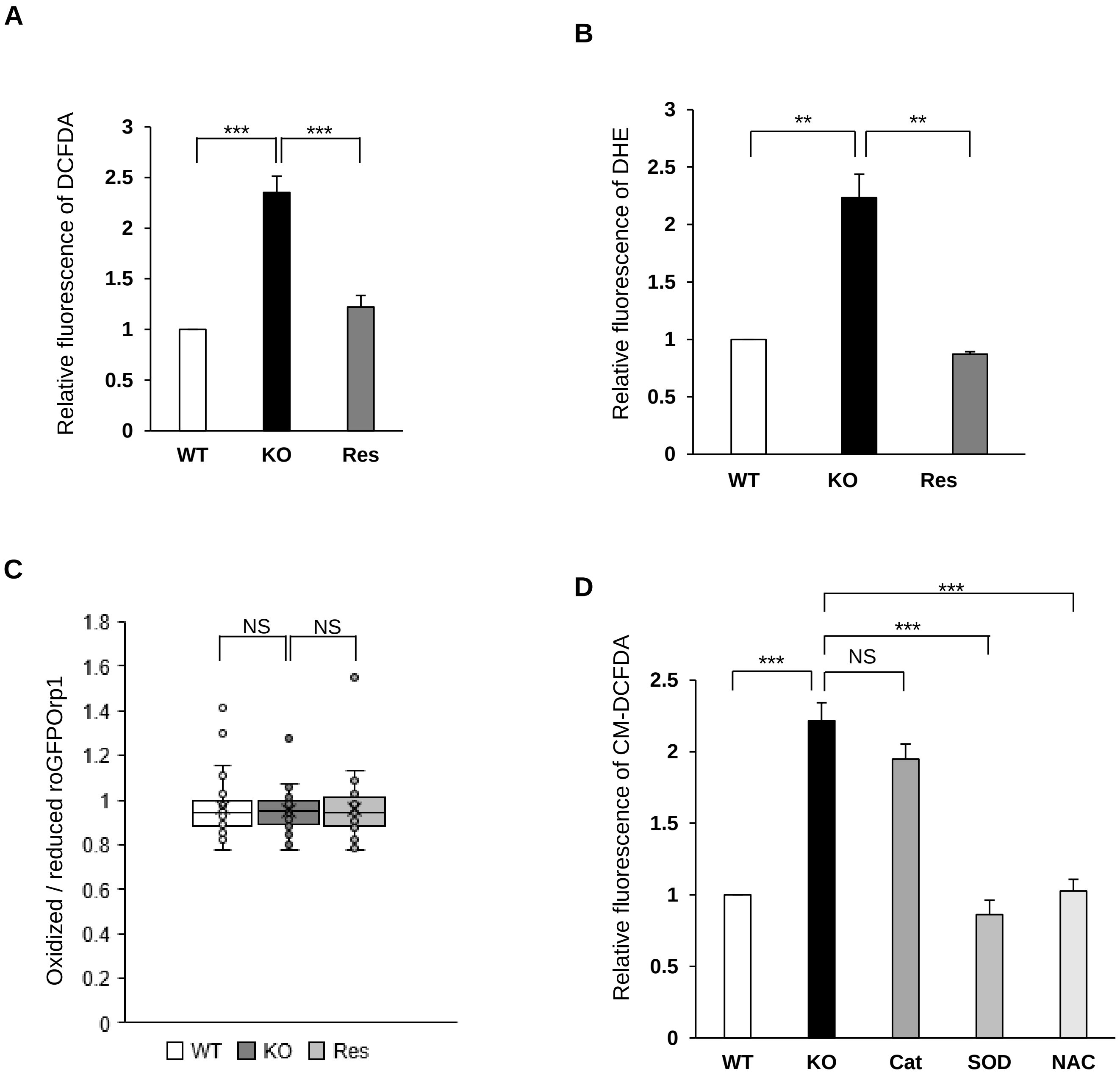

Figure S2. Quantification of intensity of ROS probes. (A) Quantification of fluorescence intensity of CM-DCFDA. (**B)** Relative intensity of DHE. **(C)** Ratio of oxidized / reduced roGFP-Orp1 probe. **(D)** Fluorescence intensity of CM-DCFDA of ROS scavenger treated knockout cells. ** and *** indicate p-value under 0.01 and 0.001, respectively.

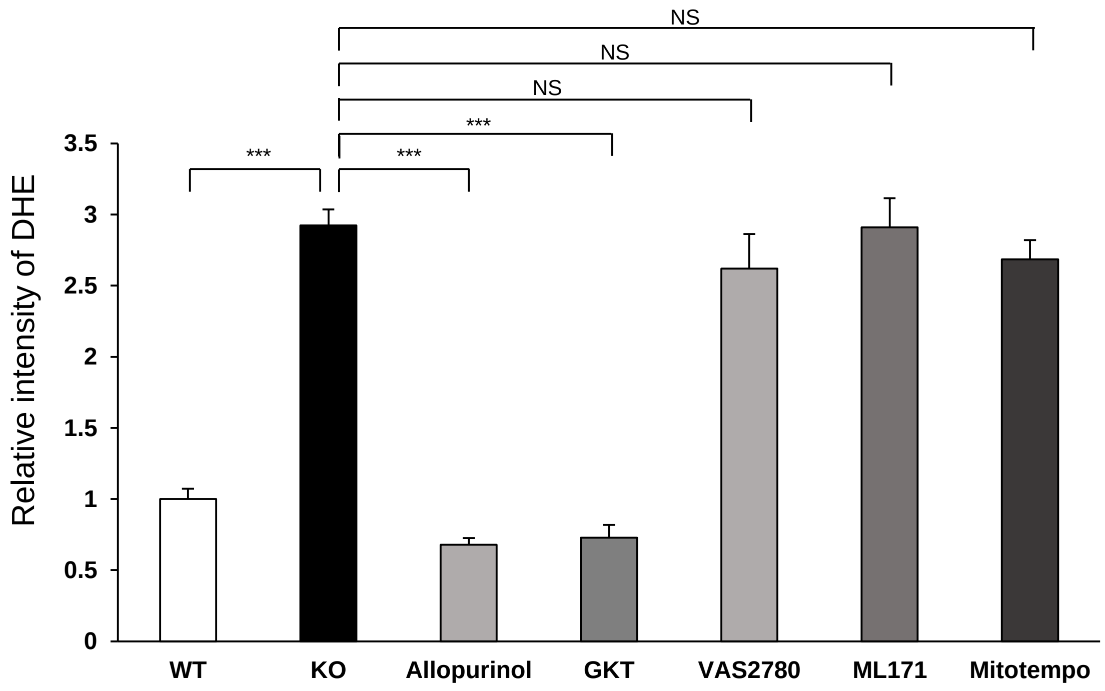

Figure S3. Quantification of superoxide accumulation in scavenger and selective inhibitor treated cells. Relative DHE signal intensities. NS and *** designate non-significant and p-value<0.001, respectively.

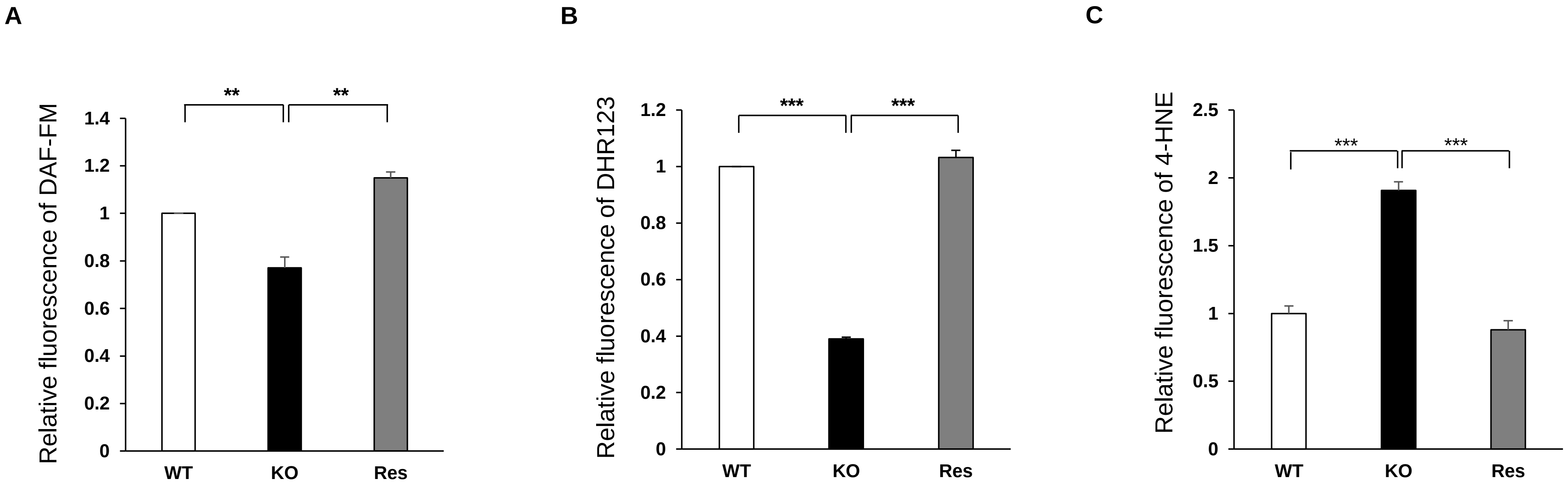

Figure S4. Measurement of signal intensities of RNS probe and 4-HNE. (A) Relative fluorescence intensity of DAF-FM signal. **(B)** Relative fluorescence intensity of DHR123. **(C)** Relative fluorescence intensity of 4-HNE.

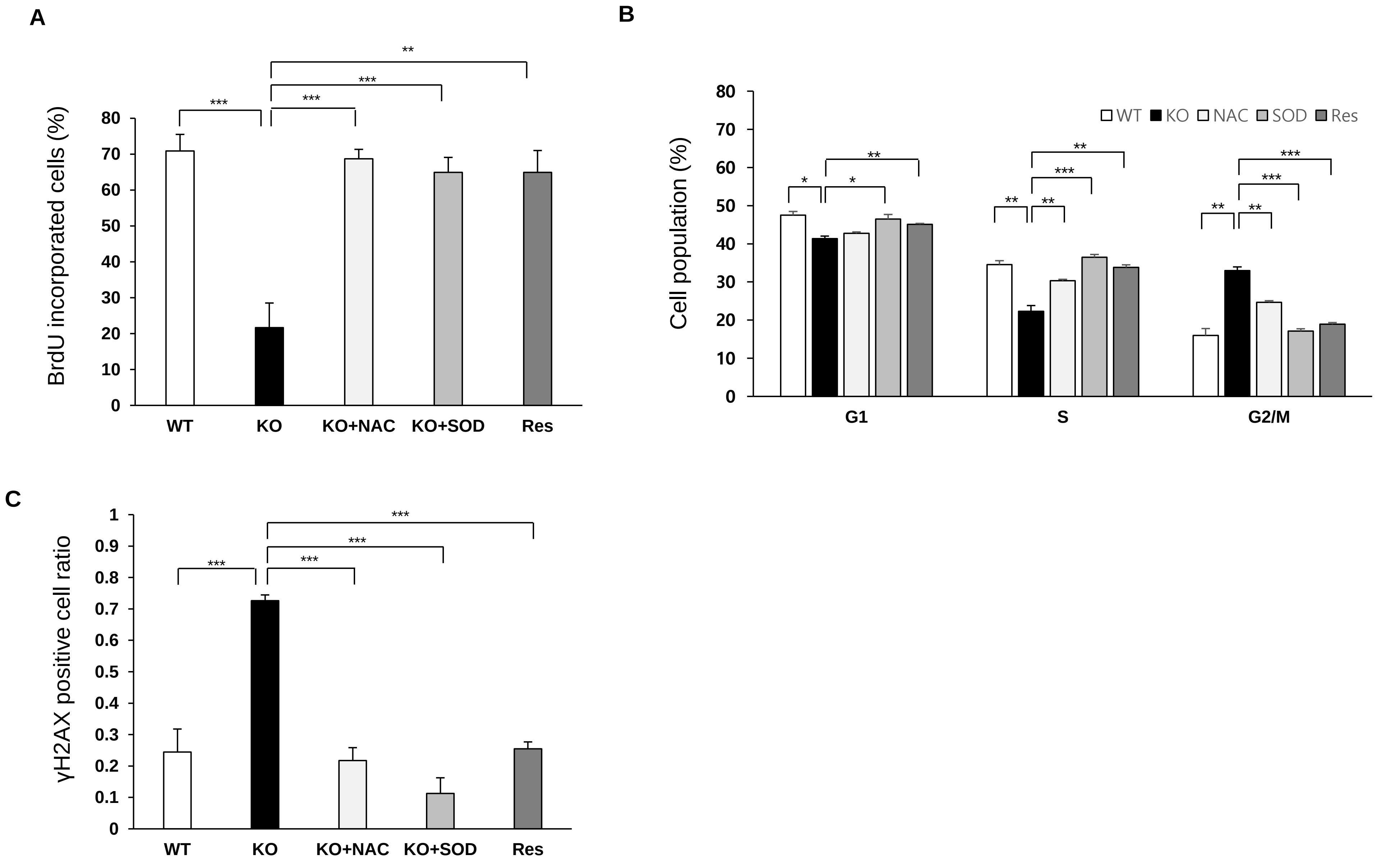

Figure S5. Analysis of cell proliferation. (A) Percent of BrdU incorporated cells. **(B)** Proportion of cell cycle. **(C)** Relative fluorescence intensity of gamma H2AX. *, **, *** indicate p-value under 0.05, 0.01, 0.001, respectively.

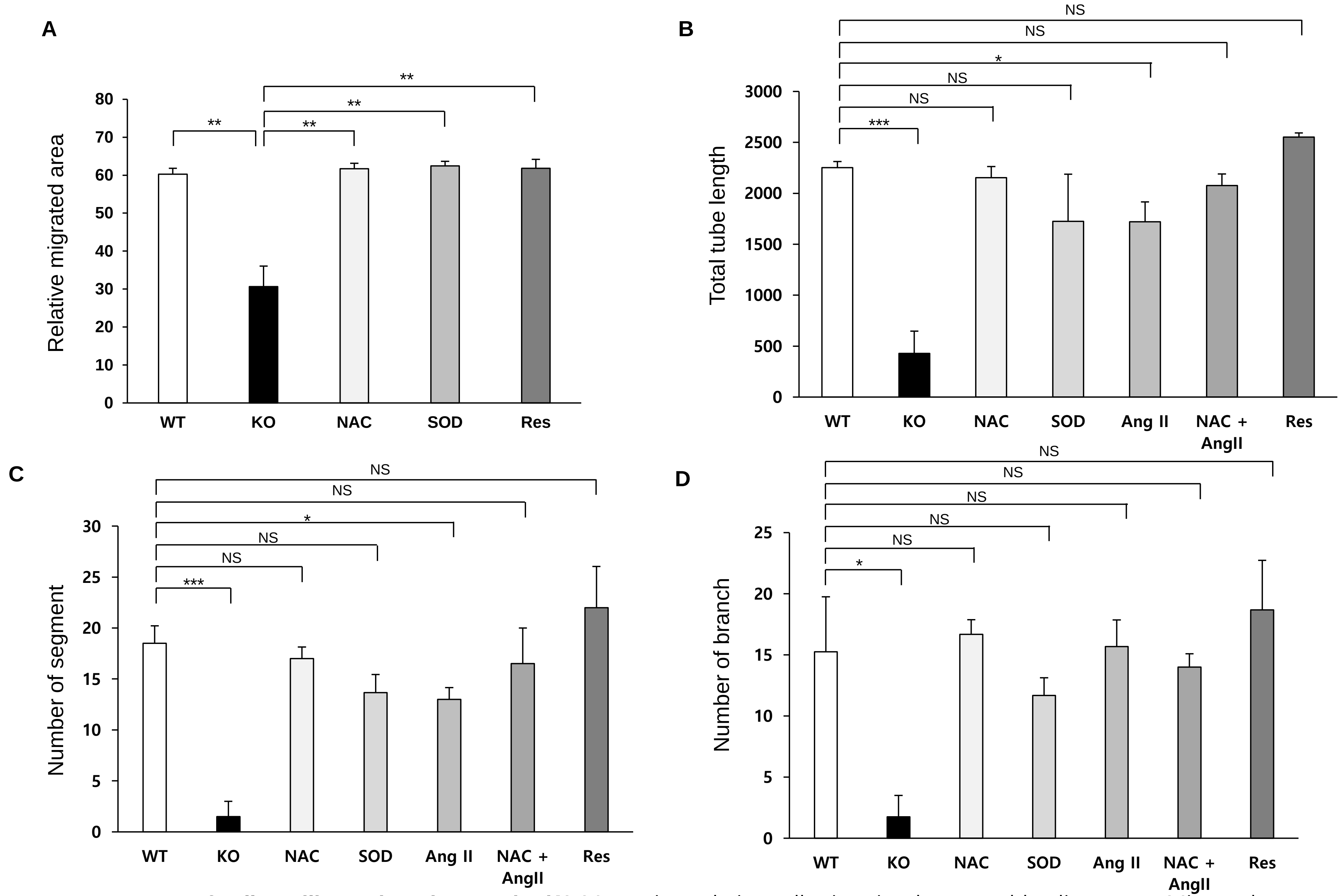

Figure S6. Measurement of cell motility and angiogenesis. (A) Measuring relative cell migration by wound healing assay. Migrated area was measured by using ImageJ as described in Materials and Methods. **(B-D)** Number of total tube length **(B)**, segment **(C)**, and branch **(D)** was measured as described in Materials and Methods. *, **, *** designate p-value <0.05, 0.01, 0.001, respectively.

Table S1. Primer sequences used in this study.

Primer Name	Sequence (5'-3')
Hprt F	AGTCCCAGCGTCGTGATTAG
Hprt R	GTATCCAACACTTCGAGAGGTC
Sod1 F	GGAAGCATGGCGATGAAAGC
Sod1 R	AGGTCTCCAACATGCCTCTC
Sod2 F	CAATAATGTTGTGTCGGGCGG
Sod2 R	TCGGTGGCGTTGAGATTGTT
Sod3 F	GATGTTCTCCCATGTCCCGG
Sod3 R	GGAAATGGGGTGGGCGATAT
Gpx1 F	AGTTCGGACACCAGGAGAATG
Gpx1 R	GAGTGCAGCCAGTAATCACC
Catalase F	CGCTGAGAAGCCTAAGAACG
Catalase R	CCAGCGTTGATTACAGGTGA
Nox1 F	AGCTTTCTGAGTAGGTGTGCATATG
Nox1 R	TTGCAAAATGAGCAGGTGCCCCT
Nox2 F	AGTGAGCTTTCCCTGTGTCTT
Nox2 R	TGCCTTCGGTGATGTGCTTTA
Nox4 F	GTTGGGCCTAGGATTGTGTT
Nox4 R	CGGCTACATGCACACCTGA
Glrx1 F	GGGGAGCTGATGACTCGGCTGAA
Glrx1 R	AGGGGCACTGGCCATCAG
Prdx1 F	TATCAGATCCCAAGCGCACC
Prdx1 R	GCTGGACACACTTCACCATG
Gsta4 F	GATGCAAAAGGATGGACACCTG
Gsta4 R	TGGTTGCCAACGAGAAAAGC
Gpx4 F	CGTCTGAGCCGCTTAC
Gpx4 R	CCCATTTACACAGATCTTGCT
Nos2 R	CTATGGCCGCTTTGATGTGC
Nos2 R	TGGAGCACAGCCACATTGAT
Nos3 F	TGGGTTTAGGGCTGTGCGG
Nos3 R	CAATAGCTGCTCAGTGGGTGA
GADD45β F	TTGCCTCTTGGGTTCGTATC
GADD45β R	AGCCTCTGCATGCCTGATAC
